# Accuracy and safety of partial thickness femtosecond laser radial and arcuate keratotomy incisions in porcine eyes

**DOI:** 10.1186/s40662-021-00268-w

**Published:** 2021-12-01

**Authors:** E. Valas Teuma, Frank A. Bucci, Raman Bedi, Gary Gray, Mark Packer

**Affiliations:** 1 LENSAR, Inc., Orlando, FL USA; 2 Bucci Laser Vision, Institute Wilkes-Barre, Wilkes-Barre, PA USA; 3 IrisARC, Chandigarh, India; 4 Packer Research Associates, Inc., 1400 Bluebell Ave., Boulder, CO 80302 USA

**Keywords:** Partial thickness micro radial and arcuate keratotomy incisions, Curved contact patient interface, Micro radial keratotomy, Arcuate keratotomy, Femtosecond laser system with curved contact interface, LENSAR femtosecond laser system

## Abstract

**Background:**

To evaluate the accuracy and safety of micro radial and arcuate keratotomy incisions constructed by a femtosecond laser system with a curved contact patient interface in porcine eyes.

**Methods:**

Partial thickness micro radial and arcuate keratotomy incisions were constructed in porcine eyes with a femtosecond laser system and evaluated for precision of depth, quality, and consistency. Optical coherence tomography was used to determine the accuracy and precision of incision depth. Corneal endothelial safety was assessed by a fluorescent live/dead cell viability assay to demonstrate laser-induced endothelial cell loss. Quality was evaluated by ease of opening and examination of interfaces.

**Results:**

In two micro radial incision groups, intended incision depths of 50% and 80% resulted in mean achieved depths of 50.01% and 77.69%, respectively. In three arcuate incision groups, intended incision depths of 80%, 600 μm or 100 μm residual uncut bed thickness resulted in mean achieved depths of 80.16%, 603.03 μm and residual bed of 115 μm, respectively. No loss of endothelial cell density occurred when the residual corneal bed was maintained at a minimum of 85–116 µm. The incisions were easy to open, and interfaces were smooth.

**Conclusions:**

A femtosecond laser system with curved contact interface created precise and reproducible micro radial and arcuate keratotomy incisions. Accuracy and precision of the incision depth and preservation of endothelial cell density demonstrated the effectiveness and safety of the system.

## Background

Advances in surgical technique, intraocular lens (IOL) technology and biometry, and the availability of increasingly accurate IOL power calculation formulas have significantly improved visual and refractive outcomes following cataract surgery [1]. Nevertheless, unintended ametropia after cataract surgery remains relatively common. According to the European Registry of Quality Outcomes for Cataract and Refractive Surgery (EUREQUO), including data on over 2 million cataract cases, only 75.1% achieve a spherical equivalent refraction within ± 0.5 D of emmetropia [2]. Therefore, even with present day cataract surgery, a significant proportion of patients are left with residual refractive error that negatively affects uncorrected visual acuity and makes them more likely to remain spectacle dependent [3].

For patients seeking complete spectacle independence, additional enhancements may be necessary for correcting residual refractive errors [4]. Among the currently available modalities, most preferred methods include corneal based procedures like laser-assisted in situ keratomileusis (LASIK), photorefractive keratectomy (PRK) and small incision lenticule extraction (SMILE) for low to moderate residual errors. Lens-based procedures like IOL exchange and piggyback IOLs may also be performed to treat refractive surprises [5].

While LASIK and PRK are safe and effective options for the correction of residual refractive error, these procedures may compromise the ocular surface [6]. Most patients undergoing cataract surgery are older in age and have concomitant ocular surface disease [7]. In addition, risks associated with corneal refractive procedures include LASIK-induced neurotrophic epitheliopathy, flap related issues, and diffuse lamellar keratitis [8]. Postoperative pain and risk of corneal haze after PRK may lead to slow visual recovery and dissatisfaction [9].

Thus, for the management of postoperative refractive error, alternative cost-effective treatment modalities that are minimally invasive can be performed soon after cataract surgery, allow rapid visual recovery, do not exacerbate dry eye, and can be used with presbyopia correcting IOLs are desirable. Corneal relaxing incisions including radial (RK) or arcuate keratotomy (AK) incisions represent one such modality. Femtosecond laser assisted AK incisions are frequently used for astigmatism correction during or after cataract surgery and have been found to be more accurate than their manual counterparts with various laser systems [10–15].

Prior to excimer laser procedures, RK was the most widely used treatment option for the correction of myopia [16]; however, structural weakening of the cornea, particularly due to deeper and longer incisions, was found to be associated with complications such as overcorrection of the initial refractive error, diurnal variations in vision, and hyperopic shift [17, 18]. Other reported complications of manual RK have included corneal perforation, incorrect incisions, epithelial defects, glare, keratitis, endophthalmitis and traumatic rupture [19, 20]. To avoid such complications, Lindstrom modified the standard RK procedure to develop a mini-RK procedure in which the length and area of incisions were reduced [17]. This mini-RK procedure was found to be safe and effective for myopia correction (albeit for lower powers) with fewer complications than the standard RK procedure [17]. Recently, the mini-RK procedure has been further modified into micro-RK by one of the authors (FAB) who has reported that the procedure is safe and effective with stable outcomes given the small size of the incisions and preservation of the central 5 mm of the cornea. The procedure can be performed as early as 3 weeks after cataract surgery [21].

Given the demonstrated improvement in the effectiveness of AK incisions constructed with the femtosecond laser as compared to manual incisions, it may be expected that RK incisions created with the femtosecond laser will further improve the efficacy of the micro-RK procedure for the correction of residual myopia. The present laboratory study was undertaken to evaluate the accuracy and safety of femtosecond laser micro-RK and AK incisions.

## Methods

In this porcine eye study, the accuracy and safety of femtosecond laser partial thickness corneal incisions including micro-RK and AK were assessed. Fifteen freshly enucleated porcine eyes were obtained from a local slaughterhouse and study procedures were performed within 12 h of enucleation. The study was conducted in compliance with the applicable requirements of good laboratory practice.

### System design

A novel curved contact lens integrated with a standard liquid optic interface was designed to allow construction of radial and arcuate partial thickness corneal incisions with the LENSAR femtosecond laser system (LENSAR, Inc., Orlando, FL). The curved contact lens constrains the corneal surface from moving in the vertical (Z) direction during the laser treatment and ensures that the first refracting surface is effectively spherical, while the liquid optic interface reduces the differences in refractive index among the media traversed by the light rays as they travel to the focal point.

The new interface model has a clear aperture of 12.75 mm in diameter, which is larger than the 11.3–11.75 mm of the average apparent limbus diameter, to facilitate centration during attachment to the eye [22]. The concave radius of curvature for the contact lens surface in contact with the cornea is 9.5 mm, which is a little larger than the nominal 7.8 mm radius of curvature of the cornea [23]. This radius of curvature was chosen to take into account corneal curvature variability from patient to patient, and so that the cornea vertex is the first point to meet the lens at its center where potential residual bubbles are pushed out with the tear film on the corneal surface as the interface is affixed. The outer diameter of the base is 21.15 mm within the opening of the palpebral aperture [23].

Customizable parameters for micro-RK incisions include depth, optical zone size ≥ 5 mm, radial length, and meridional position; for AK incisions, the parameters include depth, optical zone, arc length and meridional position. Cyclotorsion can be measured and corrected with the LENSAR laser system using iris registration based on wireless transmission of preoperative diagnostic information [24].

**Fig. 1:**
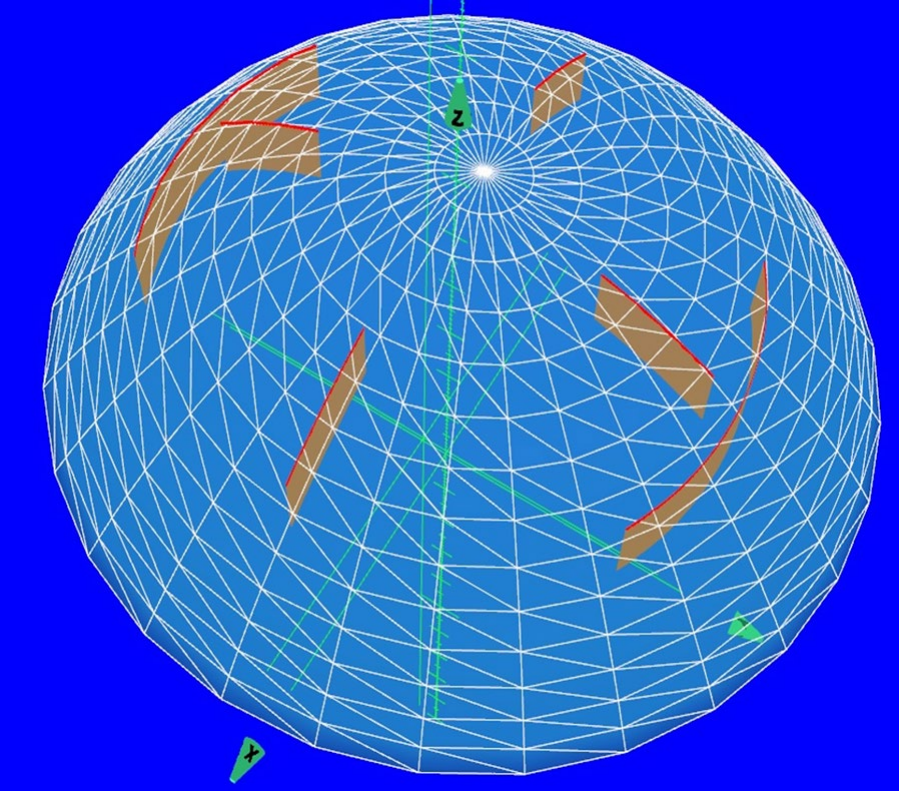
The three-dimensional (3D) computer-aided design (CAD) model reconstructed from Scheimpflug imaging by integrated software guides laser application to tissue. In this example, a combination of 4 micro-RK and a pair of AK incisions is shown positioned in the 3D reconstructed corneal model

**Fig. 2:**
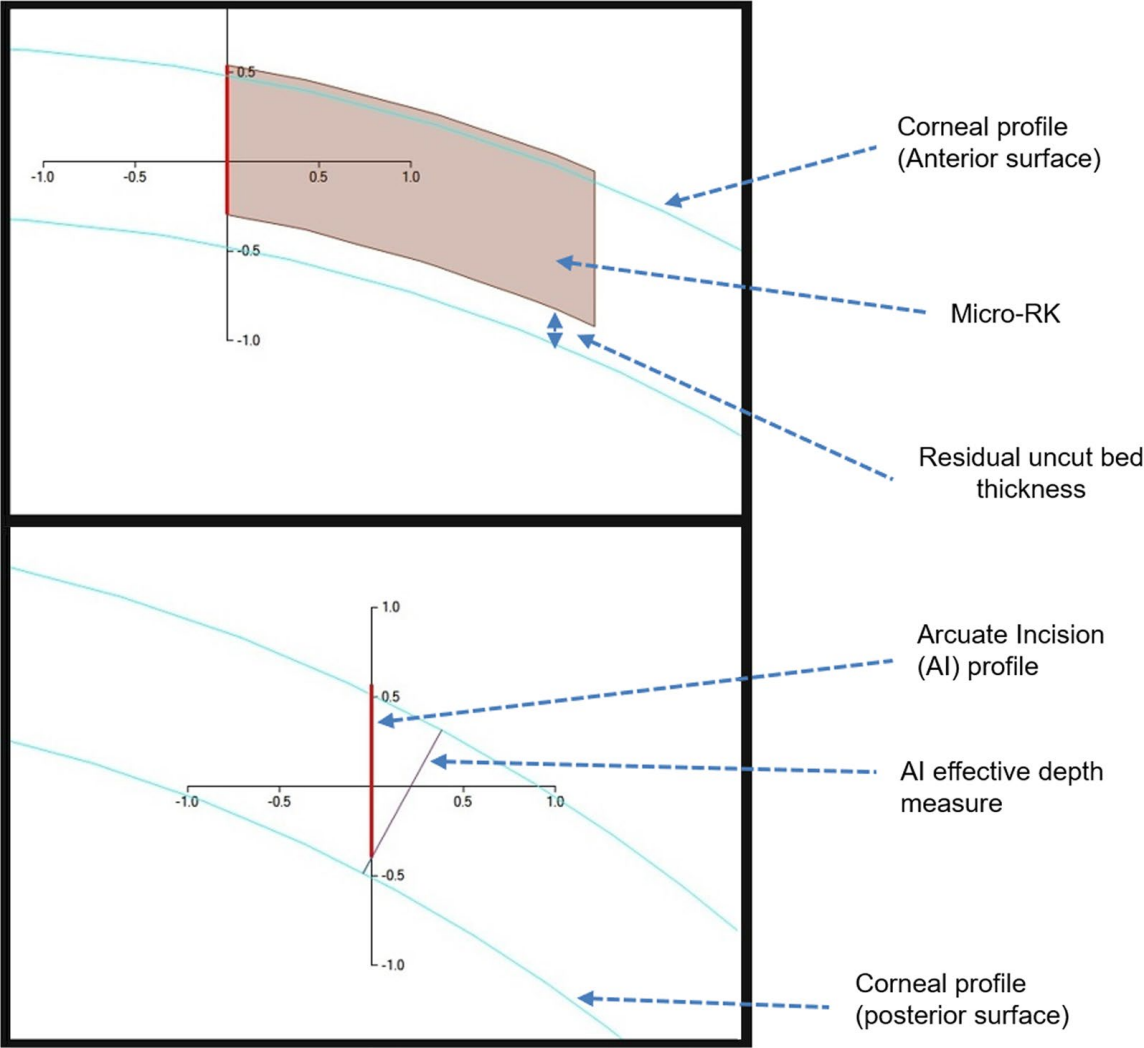
Schematic diagram of the micro-RK (top) and AK configurations (bottom) in a corneal profile

Evenly placed micro-RKs and/or paired or unpaired AKs centered on the corneal vertex were constructed perpendicular to the coronal plane of the eye as shown in Fig. 1. Schematic representations of the micro-RK and AK configurations are provided in Fig. 2. Incisions were constructed using the geometric parameters listed in Table 1.

**Table 1:** Geometric characteristics of partial thickness corneal incisions in porcine eyes

Parameters	Micro-RK	AK
Optical zone (mm)	5	10
Radial length (mm)	2	N/A
Arc length (°)	N/A	60
Incision depth/residual corneal bed thickness	25%, 50% and 80% of corneal thickness	80% of corneal thickness, 600 µm fixed depth, 100 µm residual uncut bed thickness

*N/A* not applicable; *RK* radial keratotomy; *AK* arcuate keratotomy

### Porcine globes

**Fig. 3:**
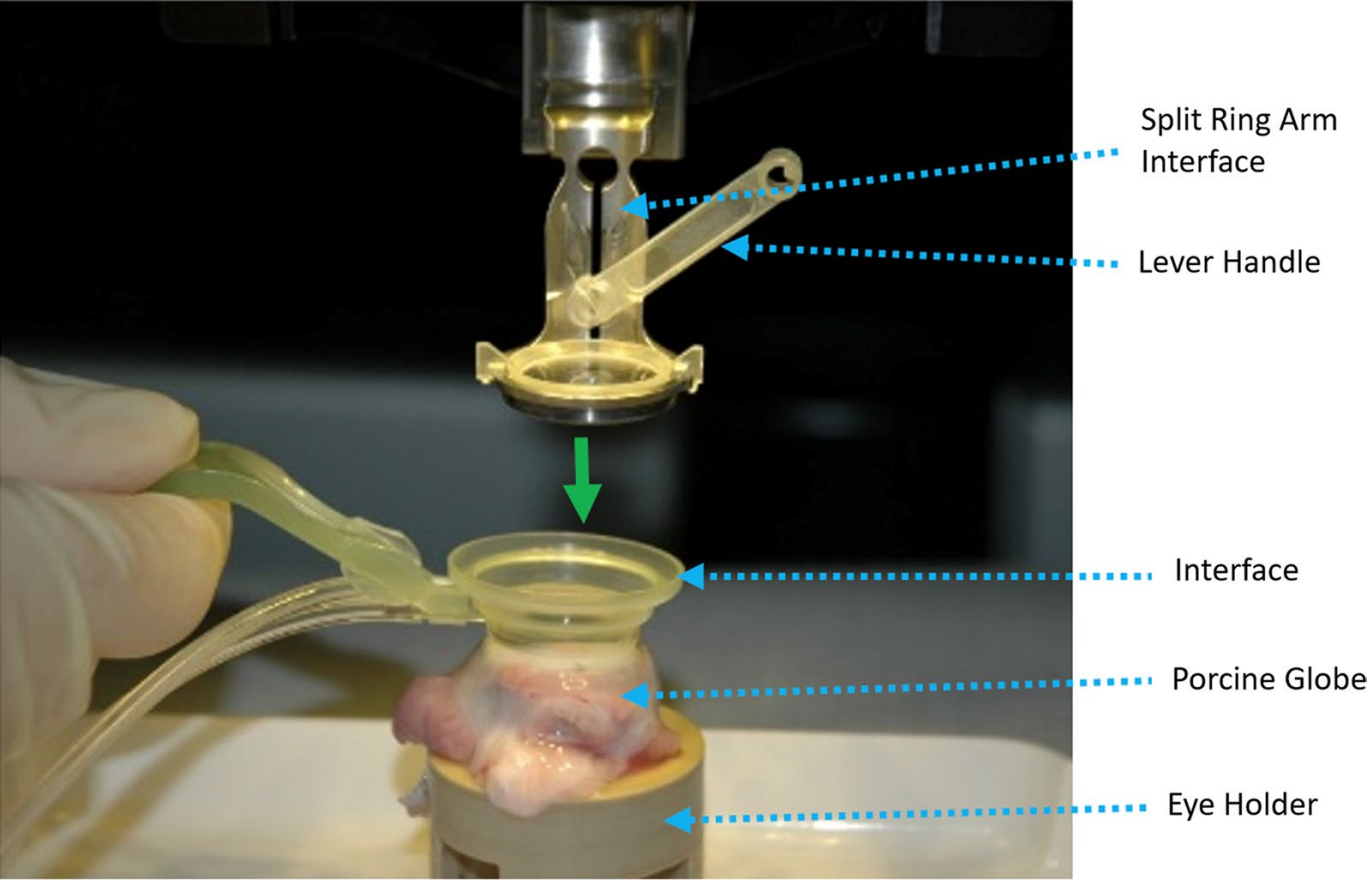
Experimental setup showing interface with irrigation and suction tubing resting on porcine globe

Freshly harvested porcine globes were placed in a customized eyecup fixture. The globes were used as they were received, except for trimming the attached connective tissue from the outside of the globe to allow placement within the eyecup. A suction ring was attached to the globe and the assembly was moved into position under the LENSAR ring arm which is the upper part of the LENSAR patient interface device (PID) assembly attached to the LENSAR laser system. The liquid chamber of the PID was filled with phosphate-buffered saline (PBS) and then the system platform was lowered until the PID ring arm docked with the suction ring and the image of the anterior corneal surface reached the upper limit indicated on the display. A photograph of the experimental set up is provided in Fig. 3. Location of the anterior and posterior corneal surfaces was determined by Augmented Reality 3-D Reconstruction. Laser parameters included line spacing of 4 µm and shot spacing of 6 µm with energy of 4.5 µJ for micro-RK and 5 µJ for AK. A laser pulse repetition rate of 80 kHz was used to generate the incisions. Micro-RKs were cut to completion prior to AKs. LENSAR’s biometric measurement and beam guidance compensated for variations in pachymetry to maintain a constant incisional depth, percentage corneal thickness or residual uncut bed thickness.

Following incision construction, eyes were transferred to the surgical microscope. A Sinskey hook (Storz Ophthalmic Instruments) and Buratto oval spatula (Duckworth & Ken Ltd. Surgical Instruments) were used to open the incisions. The ease of opening was evaluated and documented.

**Fig. 4:**
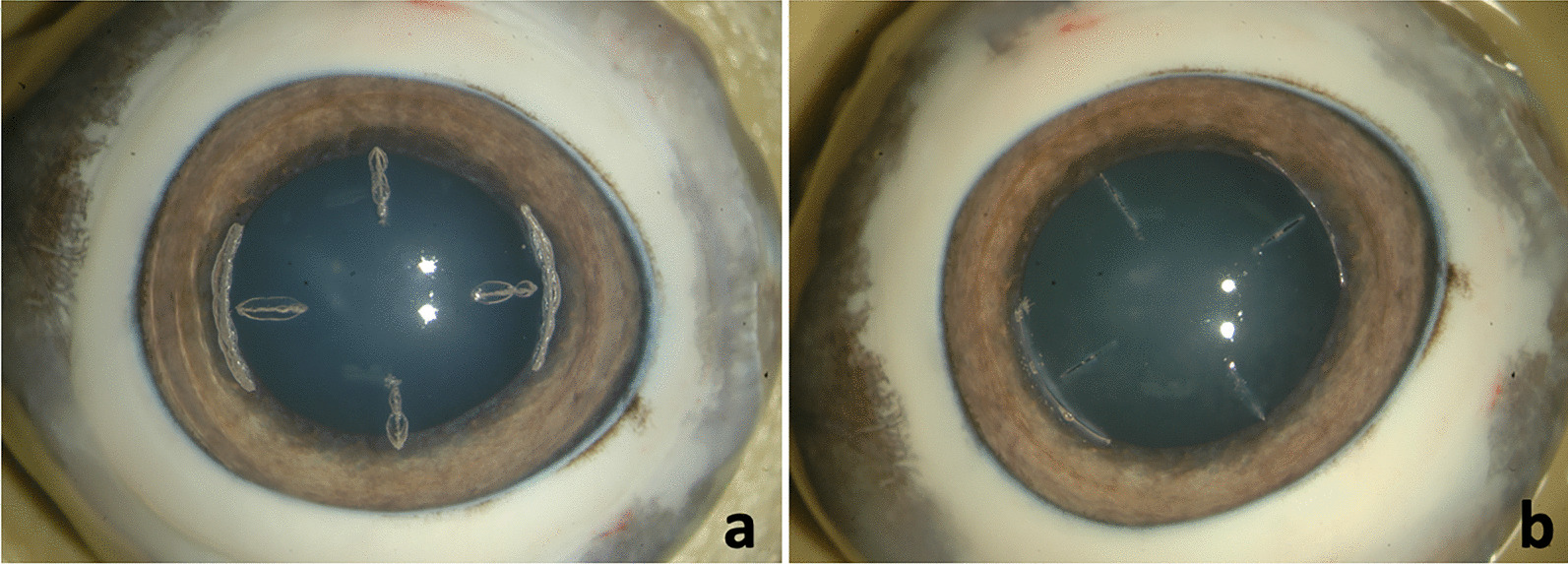
Centration and placement of 4 micro-RKs with a pair of AKs generated on porcine eyes: **a** before opening the incisions and **b** after the incisions were opened

The incision depth configurations investigated in this study (five eyes each) consisted of two types of micro-RKs (with the corneal incision depth of 50% and 80%) and three types of AKs (with 80% corneal thickness depth; fixed thickness depth of 600 µm and fixed residual uncut bed thickness of 100 µm) (Fig. 4).

### Incision measurements

**Fig. 5:**
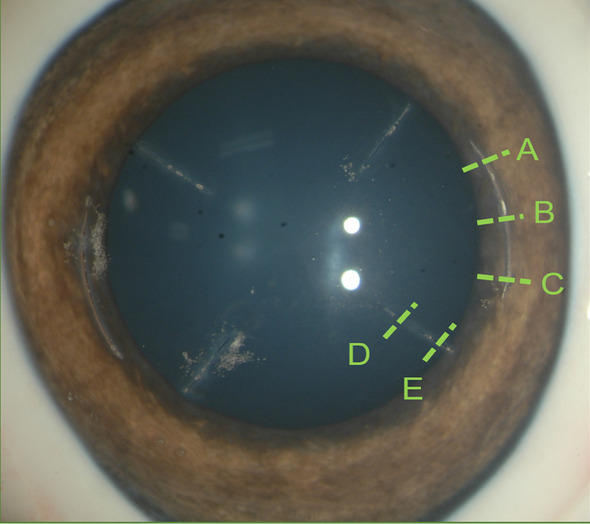
Measurement locations of the incision depth on the corneal incisions. Locations A, B & C for the AK, and locations D & E for the micro-RK

Achieved incision geometry was measured using optical coherence tomography (OCT; RT-Vue, Optovue, Inc., Fremont, CA). To assess incision accuracy and consistency, OCT was used to make three measurements for AK [near the beginning (A), at the middle (B) and near the end (C) of the arc] and two measurements for micro-RK [near the beginning (D) and near the end (E) of the incision] (Fig. 5). Results were expressed as means and standard deviations of the incision depth achieved for the different configurations of micro-RK and AK.

### Endothelial cell viability

Endothelial cell damage from incision construction was analyzed as a function of the OCT-measured thickness of the uncut, residual corneal bed. A series of paired AK incisions with arc length 90˚ and decreasing depth was created in porcine corneas. A total of 22 eyes were used; 4 eyes underwent full thickness incisions, and 18 eyes underwent partial thickness incisions of increasing residual corneal bed. A fluorescent live/dead cell viability assay method was used to provide accurate measurements of the laser-induced endothelial cell loss at 1 and 48 h as previously described by Teuma et al. [25]. The method measures endothelial cell loss from the ratio of the area of laser damage, visualized using the fluorescent stains, to the area of the entire endothelium. The regions of the cornea treated by the laser were imaged by fluorescent microscopy; the fluorescent staining allowed quantitative measurement of the area of dead/damaged and live endothelial cells along the laser incision. For assessing the percentage of endothelial cell loss, tissue samples were batched into ranges of residual corneal bed thickness (0, 61–81, 86–96 and 106–116 µm). Results were expressed as mean percent endothelial cell loss at varying residual corneal bed thicknesses.

## Results

Evaluation revealed that the incisions were easy to open, and interfaces were smooth. Occasionally, there were marginal tags due to tissue bridges which were easily separated using the Buratto spatula.

### Incision measurements

**Fig. 6:**
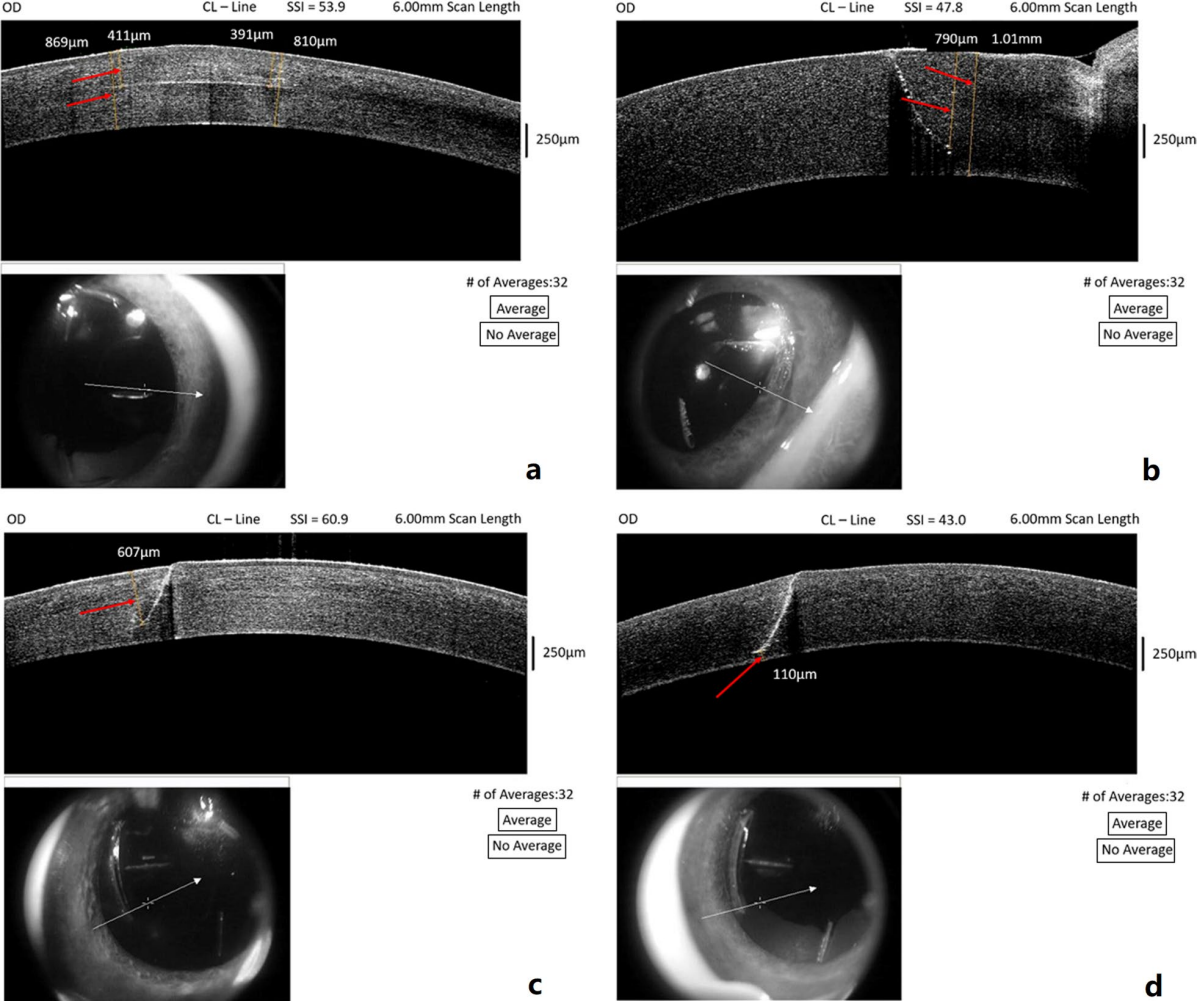
Corneal incision depth measurements: **a** percent thickness depth for the micro-RK, **b** percent thickness depth for the AK, **c** fixed incision depth for AK and **d** fixed residual uncut bed thickness. The yellow reticules next to the red arrows show the measured locations. The white arrows in the bottom OCT images indicate the location of the scan on the eye being displayed in the top image

Figure 6 shows representative images of corneal incision depth measurements. In two micro radial incision groups, intended incision depths of 50% and 80% resulted in mean achieved depths of 50.01 ± 1.72% and 77.69 ± 1.78%, respectively. Mean absolute error of intended versus achieved depth was 12.66 ± 8.25 μm in the 50% group and 23.33 ± 14.77 μm in the 80% group (Table 2).

**Table 2:** Intended and achieved corneal incision depth in porcine eyes that underwent micro-RK

Intended corneal incision depth	Number of measures	Achieved micro-RK corneal incision depth			MAE (intended-achieved) in incision depth ± SD (µm)
		Mean ± SD (%)	Median (%)	Range (%)	
50% of corneal thickness	40	50.01 ± 1.72	49.83	47.10–53.75	12.66 ± 8.25
80% of corneal thickness	40	77.69 ± 1.78	77.18	74.17–81.55	23.33 ± 14.77

Five porcine eyes were used in each set. Four micro-RK incisions were performed in each eye and two measurements were taken for each RK incision making a total of 40 measurements in each set

*MAE* mean absolute error; *RK* radial keratotomy; *SD* standard deviation

In the three arcuate incision groups, intended incision depths of 80%, 600 μm or 100 μm residual uncut bed thickness resulted in mean achieved depths of 80.16 ± 1.89%, 603.03 ± 23.53 μm and residual bed of 115 ± 16.84 μm, respectively. Mean absolute error of intended versus achieved depth was 15.65 ± 11.50 μm in the 80% group, 18.95 ± 11.74 μm in the 600 μm group and 20.48 ± 13.23 μm in the 100 μm residual bed group (Table 3).

**Table 3:** Intended and achieved arcuate incision depth in porcine eyes with three different treatment options

Intended corneal incision depth	Number of measures	Achieved corneal incision depth			MAE (intended-achieved) in incision depth ± SD (µm)
		Mean ± SD	Median	Range	
80% of corneal thickness (%)	30	80.16 ± 1.89	80.12	75.51–83.72	15.65 ± 11.50
600 µm fixed incision depth (µm)	30	603.03 ± 23.53	606.5	561–642	18.95 ± 11.74
100 µm fixed residual uncut bed thickness (µm)	30	115.00 ± 16.84	113.5	55–143	20.48 ± 13.23

Five porcine eyes were used in each set. Two arcuate incisions were performed in each eye and three measurements for each AK were taken making a total of 30 measurements in each set

*MAE* mean absolute error; *SD* standard deviation

### Endothelial cell viability

**Fig. 7:**
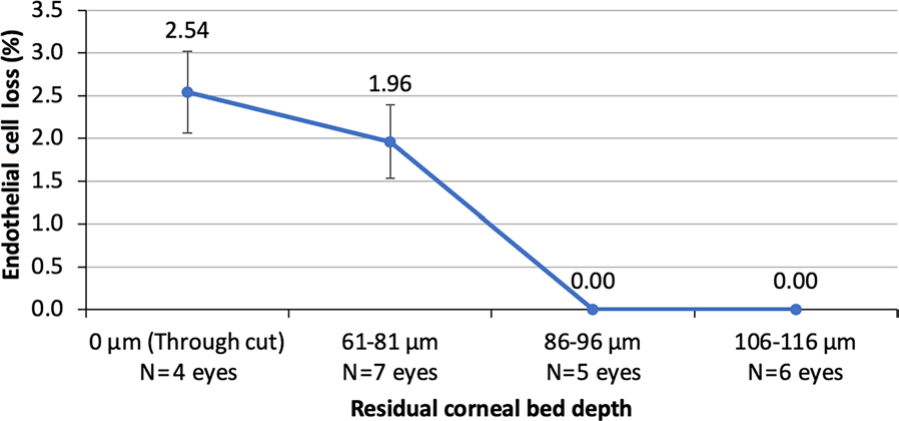
Means and standard deviations of percent endothelial cell loss for 2 × 90° subtended angle for partial thickness incisions with varying residual corneal bed depth

**Fig. 8:**
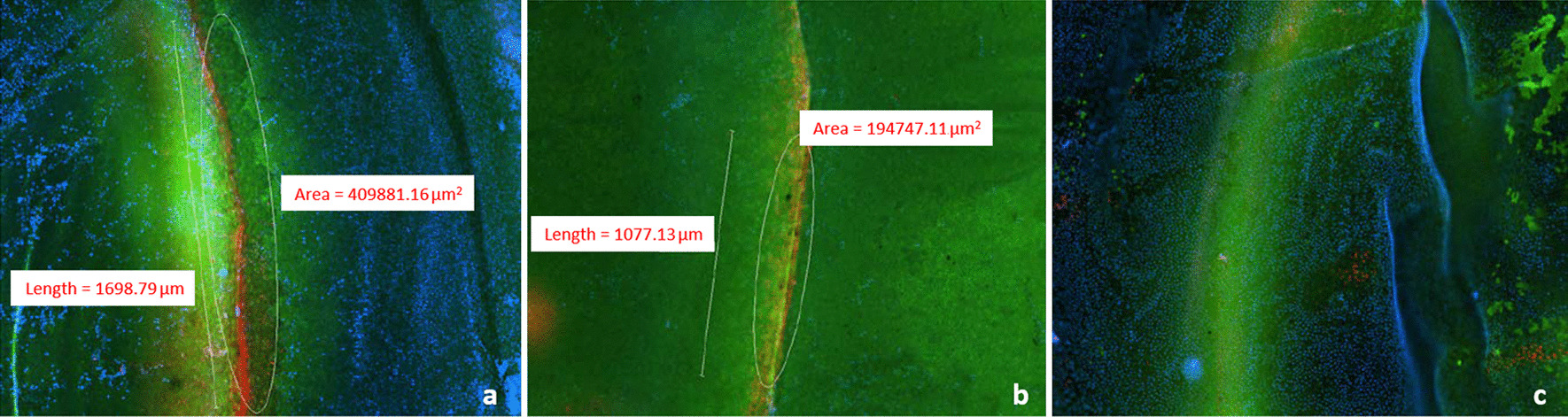
Fluorescent images depicting endothelial cell loss for PTIs of increasing residual corneal depth. Red color depicts area of damaged endothelial cells and green represents the fluorescence emitted from healthy cells. **a** Damaged/dead cells are densely distributed around the through-cut (residual corneal bed thickness 0 μm. **b** Endothelium exhibiting a lower density of damaged/dead cells as shown by the lesser extent of red fluorescence (residual corneal bed thickness 77 μm) and **c** No damaged/dead cells (residual corneal bed thickness 92 μm)

The fluorescent live/dead cell viability assay showed no loss of endothelial cells when the thickness of the corneal bed was at least 86 µm. However, at lower residual corneal bed thicknesses, the mean endothelial cell loss ranged from of 1.96% (when residual corneal bed thickness was 60–81 µm) to 2.54% (when residual corneal bed thickness was 0 µm) (Figs. 7 and 8).

## Discussion

In this study, a curved contact lens integrated with the liquid optic patient interface was used to create femtosecond laser micro-RK and AK incisions. The incisions were constructed at the intended incision depth with a high degree of accuracy and precision. Analysis of incision quality showed that the incisions were easy to open and were generated at the exact locations as programmed. The procedure appeared safe, with no loss of endothelial cell density when the residual uncut bed thickness was maintained at 86 µm or more.

Radial keratotomy has long been used for correcting myopia; however, similar to manual AK, manual RK is marred by lack of reproducibility. Complications arising due to deeper incisions include fluctuations in the cornea’s refractive power, induction of irregular astigmatism, hyperopic shift, perforation, and infection [26]. To reduce such complications, the historical RK procedure has been modified by reducing the number and length of incisions. Studies of mini-RK have found it safe and effective [17, 18, 27]. In a cadaver eye study, the intraocular pressures required to rupture the globe were comparable when comparing mini-RK and a control group without incisions, suggesting that mini-RK does not significantly weaken the cornea [18].

Mini-RK has further evolved into micro-RK in which the number of incisions has been further reduced and the length of the incisions is restricted to less than 2.5 mm, maintaining a central optical zone of 5 mm. In a recent study [21] involving 1042 pseudophakic eyes that underwent micro-RK/AK with diamond blade (532 underwent micro-RK/AK, 110 underwent only micro-RK, 400 underwent only AK), Bucci reported significant reduction in mean refractive spherical equivalent (− 0.59 D preoperatively to − 0.07 D postoperatively) as well as mean refractive astigmatism (1.03 D preoperatively to 0.24 D postoperatively), with mean uncorrected visual acuity improving from 20/38.6 to 20/23.8. In Bucci’s experience, diamond blade created micro-RK/AK incisions guided by the Lindstrom nomogram for RK incisions and modified Woodcock nomogram for AK incisions was safe and effective for the management of low to moderate pseudophakic residual myopia and myopic astigmatism without significant hyperopic shift or fluctuating vision [28]. In addition, Bucci noted several advantages of micro-RK/AK as compared with PRK, including the central 5 mm zone of the cornea remaining untouched, faster visual recovery, less postoperative discomfort, no need for bandage contact lens, less stress on the ocular surface and reduced procedure costs.

Femtosecond laser-assisted AK incisions have been shown to be more precise and accurate than manual incisions, resulting in better outcomes [12, 13, 15, 29, 30]. With femtosecond laser-assisted AK, up to 96% of eyes have been found to achieve postoperative astigmatism less than or equal to 0.5 D [10–12, 14, 31, 32]. Like femtosecond laser AK incisions, it is expected that femtosecond laser micro-RK incisions will improve precision and accuracy, leading to better predictability.

Femtosecond laser systems rely on patient interfaces for docking [33]. Different patient interfaces with and without corneal applanation procedure have been introduced [33]. Fluid filled liquid optic interfaces are widely used in femtosecond laser-assisted cataract surgery. A contact lens bonded to the silicone ring of the liquid optic interface may cause less increase in intraocular pressure and reduce subconjunctival hemorrhage. Furthermore, with less strain on the cornea, the PID is not expected to induce corneal folds [34]. Curved contact patient interfaces may be better suited for creating RK incisions as the curved contact lens constrains the corneal vertex from moving in the vertical (*Z*) direction, allowing for the incisions to be placed precisely at an intended depth within the cornea. Moreover, applanation of the cornea by the curved contact lens ensures that the first refracting surface is effectively spherical and allows precise localization of the corneal anterior surface.

Femtosecond laser-assisted AK incisions are frequently used for the correction of residual astigmatism; however, residual refractive error seldom presents as astigmatism alone and is frequently associated with myopia. The ability to make simultaneous femtosecond laser-assisted AK and micro-RK incisions adds to the surgeon’s armamentarium allowing correction of mild to moderate residual myopia as well as astigmatism. However, few studies have been presented or published regarding the effectiveness and predictability of these procedures for residual refractive error after cataract surgery. While lamellar corneal refractive surgery has been a popular modality for addressing residual pseudophakic refractive error, micro-RK and AK provide an alternative approach that offers rapid rehabilitation, minimal morbidity, and short healing time.

While laboratory studies such as the current investigation represent a prerequisite to clinical implementation of new technology, they have inherent limitations such as the inability to demonstrate refractive effectiveness in the correction of myopia and astigmatism. Nevertheless, this study provides critical data regarding accuracy of incision construction and protection of the corneal endothelium to support future clinical research.

## Conclusions

In this porcine eye study, a femtosecond laser equipped with a curved contact patient interface accurately constructed micro-RK and AK incisions with minimal endothelial damage. The use of a curved contact patient interface constrains the corneal vertex from moving in the vertical (*Z*) direction allowed for the incisions to be placed precisely at an intended depth within the cornea. Accuracy and precision of the incision depth and preservation of endothelial cell density demonstrated the ability of the femtosecond laser to accurately construct micro-RK and AK incisions. Future clinical studies will generate data necessary to demonstrate the safety and effectiveness of femtosecond laser micro-RK and AK incisions, as well as refine the refractive nomograms to optimize their outcomes.

## Data Availability

The datasets used and/or analyzed during the current study are available from the corresponding author upon reasonable request.

## Abbreviations

IOL: Intraocular lens
EUREQUO: European Registry of Quality Outcomes for Cataract and Refractive Surgery
LASIK: Laser-assisted in situ keratomileusis
PRK: Photorefractive keratectomy
SMILE: Small incision lenticule extraction
RK: Radial keratotomy
AK: Arcuate keratotomy
PID: Patient interface device
OCT: Optical coherence tomography
